# UV Light Assisted Coating Method of Polyphenol Caffeic Acid and Mediated Immobilization of Metallic Silver Particles for Antibacterial Implant Surface Modification

**DOI:** 10.3390/polym11071200

**Published:** 2019-07-18

**Authors:** Ji Yeon Lee, Ludwig Erik Aguilar, Chan Hee Park, Cheol Sang Kim

**Affiliations:** 1 Department of Mechanical Design Engineering, Graduate School, Chonbuk National University, Jeonju 54896, Korea; 2 Department of Bionanosystem Engineering, Chonbuk National University, Jeonju 54896, Korea; 3 Division of Mechanical Design Engineering, Chonbuk National University, Jeonju 54896, Korea

**Keywords:** titanium implants, polycaffeic acid, metallic silver particles, anti-bacterial properties

## Abstract

Titanium implants are extensively used in biomedical applications due to their excellent biocompatibility, corrosion resistance, and superb mechanical stability. In this work, we present the use of polycaffeic acid (PCA) to immobilize metallic silver on the surface of titanium materials to prevent implant bacterial infection. Caffeic acid is a plant-derived phenolic compound, rich in catechol moieties and it can form functional coatings using alkaline buffers and with UV irradiation. This combination can trigger oxidative polymerization and deposition on the surface of metallic substrates. Using PCA can also give advantages in bone implants in decreasing inflammation by decelerating macrophage and osteoclast activity. Here, chemical and physical properties were investigated using FE-SEM, EDS, XPS, AFM, and contact angle. The in vitro biocompatibility and antibacterial studies show that PCA with metallic silver can inhibit bacterial growth, and proliferation of MC-3T3 cells was observed. Therefore, our results suggest that the introduced approach can be considered as a potential method for functional implant coating application in the orthopedic field.

## 1. Introduction

In the orthopedic field, peri-implant infection is one of the most serious complications leading to 14% of all implant failures. It can lead to prolonged hospitalization, financial burden, and even death [1,2]. Infection at the periphery of implants is usually due to two reasons: The attachment and formation of biofilm by microorganisms through colonization and the compromised immune ability at the interface of the implant and tissue [3,4,5]. Microorganisms are able to attach to the implant, especially biocompatible implants due to protein membrane formation [6,7,8,9]. Usually, the human body is able to defend itself from infections, depending on the immune response system [10,11]. However, in order to prevent infection during and after the placement of implants, stringent aseptic surgical conditions are used, and antibiotics are administered. However, these approaches have obvious shortcomings, and research has thus been carried out on finding a way to modify implanted materials at the surface level. Therefore, in order to avoid the formation of biofilm, which ultimately prevents infection, the antibacterial properties of the materials used in implants need to be increased.

Currently, titanium (Ti) alloys are one of the most common materials used in many biomedical applications such as dental and orthopedic implants because of its biocompatibility, resistance to corrosion, high strength-to-weight ratio, good tolerance, and presence of reactive titanium oxide surface layer [12,13,14,15,16]. Since titanium was first used for implant fabrication in 1930, it has been shown to be superior to stainless steel or cobalt alloys. Titanium (Ti) and its various alloys, which are mixed with vanadium or aluminum, were first used in surgery in 1950. Because of its mechanical properties (modulus and hardness), Ti alloys play an important role in orthopedic surgery and have been used continuously since these properties were first revealed [17,18,19]. Moreover, the stiffness of Ti actively adjusts bone cell phenotypic specification [20,21,22].

Despite the excellent physical properties of titanium, infection is still a major issue. To address this, several studies have attempted to improve the antibacterial capability of titanium via surface modification and coating using functional materials such as polymeric coating [23,24,25]. Many studies have explored several surface coatings, examples of which are the use of catechol-containing moieties such as multiple 3,4-dihydroxyphenylalanine (DOPA) derived from mussels, which strongly binds to proteins and is capable of surface coating functionalization, and the development of a special wetting surface [26,27,28]. In our study, we used poly-caffeic acid (PCA), which is a naturally derived phenolic compound that can form a multifunctional coating on various substrates by polymerization under a mild alkali condition. When used as a biomedical applied coating material, PCA is inherently better than polydopamine due to its various properties such as antioxidant, anticancer, anti-inflammatory, antihypertensive, and antibacterial agent. It is, therefore, a promising candidate material for surface coating on titanium implants [29,30,31,32].

Phenolic molecules like polycaffeic acid are also proven to have in situ reduction properties and are widely used for the green synthesis of metallic nanomaterials especially in the formation of metallic silver [33,34,35]. Metallic silver is known as an antibacterial agent, and several hypotheses have been presented about its mechanisms in inhibiting bacterial growth [36,37,38,39]. In this current study, we utilized the inherent property of PCA as a reduction agent to reduce Ag ions to metallic Ag particles, in order to create a multifunctional surface to mitigate the occurrence of bacterial infection in metallic implant procedures. Several theories have been presented on the antibacterial effects of Ag [40,41]: (1) Ag adheres to the bacterial cell wall and cause a structural change in the bacterial cell, which induces cell death. Alternatively, Ag comes into contact with the bacteria so that the action of the free radicals on the Ag causes holes in the cell, which then dies. (2) Ag emits silver ions that bind to the thiol groups and cause damage to the cell. (3) Silver is weakly acidic and naturally reacts with cells that are slightly alkaline due to the characteristic of acid to react with the alkali, eventually leading to the death of the bacterial cells. (4) Ag that enters into the bacteria affects the signal transduction due to the phosphorylation of the protein substrate, which inhibits signal transduction and induces growth arrest. Here, we focus on the prevention of peri-implant infection by coating on a titanium surface using a combination of two different antibacterial materials for a synergistic enhancing effect of anti-inflammation using a simple method. This work will explore the potential application of this approach in the orthopedic field.

## 2. Materials and Methods

### 2.1. Materials

Ti foil (0.127 mm thick, annealed, 99%) was obtained from Alfa Aesar (Daejon, Korea). The foil was cut into small pieces with dimensions of 2.5 cm × 5 cm. Silver nitrate (AgNO_3_, MW 169.87 g/mol) was purchased from SHOWA chemicals (Gyoda, Japan). Chemical products such as Caffeic Acid (CA) (3,4-Dihydroxybenzeneacrylic acid, MW 180.16 g/mol), and sodium hydroxide (NaOH, MW 40.00 g/mol, pellets (anhydrous)) were purchased from Sigma Aldrich Chemical Co. Ltd. (Seoul, Korea) and used without further purification. All aqueous solutions were prepared by ultrapure water purified using a Milli-Q UV-Plus water purification system (Millipore, Bedford, MA, USA). The resistivity of the water was >1018 MΩcm^−1^.

### 2.2. Preparation of the Polycaffeic Acid Coated-Metallic Silver Functionalized Titanium Samples

**Figure 1:**
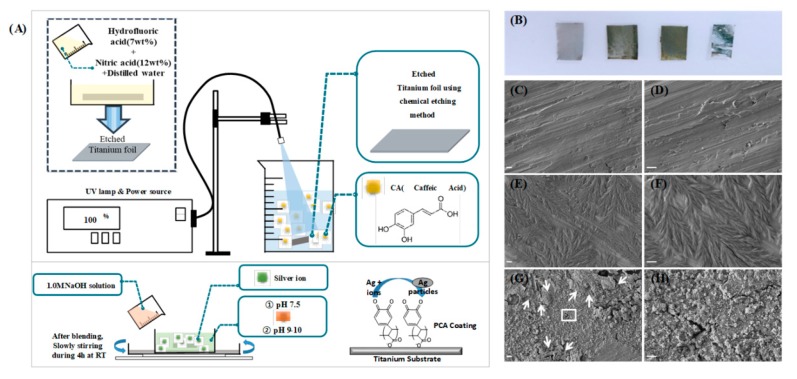
(**A**) Schematics of the process of coating polycaffeic acid (PCA) on etched titanium (upper image) and synthesis of metallic silver on the Ti–PCA substrate (lower image) and (**B**) photo and (**C**–**H**) FE-SEM image in two magnitudes (scale bar X 5.0 k = 1 µm, 20.0 k = 5 µm) of titanium foil according to each process.

Each Ti piece cut to the same size was chemically etched to remove the passive oxide layer and impurities on the surface. The Ti pieces were then submerged into a solution of hydrofluoric acid (7 wt.%) and nitric acid (12%) in distilled water (DW, 81 wt.%) for approximately 20 s followed by instant sonication first in ethanol and then in DW for ten minutes. These Ti pieces were then dried under a nitrogen atmosphere. As shown in Figure 1A, after soaking, one of the prepared Ti pieces was submerged into the CA solution (0.09 g of CA powder with 30 mL of 0.1 mM pH10 carbonate-bicarbonate buffer solution) under UV irradiation (200–300 nm wavelength) at 95% power setting using Omnicure^®^ Series 1500 lamp for 4 h (distance between top of the solution and tip where light is emitted is 5 cm) with slow stirring at room temperature. For the metallic silver in-situ immobilization using a polyphenol reduction method, the Ti pieces after coating with PCA were fully submerged in a silver nitrate solution (20 mL of 4 mM AgNO_3_ solution with DW (which has a pH of 8.5–9.0) using 1 M NaOH solution) for 24 h at room temperature with continuous stirring. The samples were then washed again in DW and air-dried at room temperature.

### 2.3. Characterization of Surface Properties

The morphology of the coating was observed using field emission scanning electron microscopy (FE-SEM, SUPRA 40VP, Carl Zeiss, Germany) at accelerating voltages of 15.0 and 5.0 kV. The samples were sputter-coated under argon to render them electrically conductive. The surface topography of the prepared samples was visualized using atomic force microscopy (AFM, Multimode-8, Bruker, Germany) operating in tapping mode with a scan rate of 0.7535 Hz. Energy dispersive X-ray spectroscopy (EDS) coupled with FE-SEM was employed to analyze the elemental composition of the specimens′ surface. Elemental mapping was then performed to confirm the distribution of elements on the surface of each sample. X-ray photoelectron spectroscopy (XPS, AXIS-NOVA, Kratos Inc., San Diego, CA, USA) data were obtained with a monochromatized Al K-alpha X-ray irradiation source. To determine the surface hydrophobic-philicity of the samples, a water droplet of 5 μL was carefully dropped onto the sample′s surface, and the water contact angle was measured at 1 and 5 s intervals using a contact angle meter (GBX, Digidrop, France) at ambient temperature.

### 2.4. Determination of Antibacterial Activity

Three types of bacterial colonies were prepared to test the antibacterial property of the coating surface. Gram-negative bacteria *Escherichia coli* (*E. coli*), gram-positive bacteria *Staphylococcus Aureus* (*S. aureus*) and *Pseudomonas aeruginosa* (*P. aeruginosa*) were separately thawed in lysogeny broth (LB) and were incubated separately for 24 h in a shaking incubator maintained at 37 °C temperature. Using the spread plate method, after mixing the prepared LB solution with the thawed bacteria 1 mL of bacterial suspension containing around 10^5^ colony forming units for each bacteria, the same amount of bacterial solution was suspended on the nutritive agar plate, and the samples were carefully placed on the inoculated plates. After spreading the bacteria on the plate, three different types of titanium samples (etched Ti, Ti–PCA, and Ti–PCA–Ag) were placed on these plates in a triangular orientation and incubated at 37 °C for 24 h. The titanium samples were sterilized under ultra-violet (UV) radiation for twelve hours before placing on agar Petri plates. All specimens were tested for antibacterial activities using the growth inhibition study method, which involves measuring the % inhibition area (mm) of no bacterial growth around each sample. All antibacterial activity tests were performed in triplicate (*n* = 3 per group per type of bacteria).

The % inhibition was calculated using the following equation.

(1) $$\%\;\mathrm{inhibition}=\frac{\mathrm{area}\;\mathrm{inhibited}\;\left(\mathrm{mm}\right)-\mathrm{size}\;\mathrm{of}\;\mathrm{the}\;\mathrm{sample}\;\left(\mathrm{mm}\right)}{\mathrm{Size}\;\mathrm{of}\;\mathrm{the}\;\mathrm{sample}\;\left(\mathrm{mm}\right)}\times 100$$

### 2.5. Elution of Ag from the Ti–PCA–Ag Sample

To determine the amount of Ag being eluted from Ti–PCA–Ag sample, we have submerged a round sample with a circumference of 10 mm in distilled water (1 mL). The eluted Ag was then detected using inductive coupled plasma optical emission spectroscopy (ICP-OES) (iCAP-7000 qTegra, Thermo Fisher Scientific, Waltham, MA, USA). Three different time points were used on day 1, 2, and 3 (*n* = 3).

### 2.6. In Vitro Biocompatibility Study

Biocompatibility testing was conducted using a pre-osteoblast (MC-3T3) cell line obtained from normal mouse clone 4, ATCCR CRL-5293. The medium for the growth of the cell line was alpha-Minimal Essential Medium (αMEM, Sigma Chemical Co., St. Louis, MO, USA) supplemented with 10% fetal bovine serum (FBS, Sigma Chemical Co., St. Louis, MO, USA) and 1% penicillin/streptomycin (100X). The cells were cultivated in the cell culture plate (SPL Life Science, Gyeonggi-do, Korea) and placed in an incubator at 37 °C with 5% CO_2_ and 95% humidity. Smaller circular samples of 6.5 mm in diameter were cut from each sample and fixed in the wells of a 96-well cell culture plate (SPL life science, Gyeonggi-do, Korea). After sterilization under UV radiation for 3 h and washing three times with phosphate buffered saline (PBS), the cells with a density of 2 × 10^3^ cells/well were seeded on the samples by pipetting onto the center and placing into an incubator for five days. During the five days, the cell culture medium was replaced every 24 h. The cell biocompatibility of the samples was measured using a cell counting kit-8 (CCK-8) after one, three, and five days of cell culture. At the appointed time, the solution was added to each well and was incubated for 2 h in a 37 °C incubator with 5% CO_2_ and 95% humidity. The measurement of the absorbance was checked at 450 nm using a microplate reader (Tecan, Austria).

The cells were then subsequently imaged using confocal laser microscopy (Zeiss, SR CLSM, LSM 880, Carl Zeiss, Germany). The cell morphology and distribution of MC-3T3 cells on each sample (etched Ti, Ti–PCA and Ti–PCA–Ag) were evaluated after five days using super-resolution confocal laser scanning microscope (SR CLSM, LSM 880, Carl Zeiss, Germany). Beforehand, the MC-3T3 cells on samples were fixed in 4% paraformaldehyde solution overnight at 4 °C and then washed three times with 1% PBS (phosphate buffer saline). After that, the MC-3T3 cells were immersed in 0.5% Triton X-100 for 2 min at room temperature and then washed two times with 1% PBS. After blocking with 1% human serum albumin HSA/PBS dilution for 30 min, the cells in each of the samples were stained with Actin-green and DAPI for actin filament staining and cell nuclei visualization, respectively.

### 2.7. Image and Statistical Analysis

All images were analyzed using ImageJ software. The results are presented as the mean ± standard deviation (SD) for n = 3. Statistical significance was assessed by determining the level of significance with a one-way ANOVA followed by a Tukey test for means comparison using the OriginPro 8.5 software (*p* < 0.05) (OriginLab Corporation, Northampton, MA, USA).

## 3. Results and Discussion

### 3.1. Confirmation of Coating and Surface Morphology Analysis

As seen in Figure 1A, the coating process followed a simple UV assisted polymerization process of caffeic acid on the surface of the Titanium foil, the immobilization of metallic silver on the surface was also followed right after the coating of PCA on top of the Ti substrate, Ag ions can be immobilized and reduced to metallic silver particles in situ by using the catechol moiety of the PCA [4]. It has been recently discovered that natural polyphenols can be deposited via electromagnetic radiation (UV-light) to various substrates like glass and stainless steel [26]. After the UV-light irradiation of caffeic acid over the titanium substrate, the apparent difference in color among the four Ti pieces was observed, as shown in Figure 1B. Visual inspection initially indicates that the coating was successfully deposited on the surface of the titanium. The leftmost pure Ti specimen has a matte light gray color, while the Ti that was chemically etched has a darker color due to the removal of the oxide layer on its surface. In the case of the Ti specimen coated with PCA, the material developed a visible yellowish-gray coating over the Ti specimen. Unlike the previous test piece, the surface of the specimen with metallic silver on the PCA coating is shiny rather than matte.

In order to confirm the coating on the surface of the Ti samples, we employed field emission scanning electron microscopy and the FE-SEM images in Figure 1C–H shows the surface morphology of each Ti sample in different conditions. Etched Ti samples are shown to be featureless (Figure 1C–D), while in the case of the Ti–PCA specimen, the polymerized caffeic acid (PCA) formed leaf-like structures and covered almost the entire specimen (Figure 1E–F). The PCA coating was formed by gathering several thousand strings of strands of about 1 μm in length. In Figure 1G–H, the specimen is covered with metallic Ag particles with traces of the PCA remaining after the process (indicated by arrows). The Ag reduction properties of PCA are further shown in the FESEM images as the particles had been deposited in situ. The potential of catechol moieties as a redox agent has been explored and the overall mechanism for reducing Ag ions to metallic Ag is generally through the formation of quinone and semiquinone in the polycaffeic acid chemical structure at pH levels higher than 9.45. The highly electronegative oxygen in the quinones can attract and stabilize the Ag ions and form metallic silver on the surface of the polycaffeic acid coating.

Another novel feature of this method is the use of plant-derived polyphenol. Caffeic acid has been shown to have antibacterial, antiviral, anti-cancer, and anti-inflammatory effects [42,43,44]. In comparison to polydopamine, coatings based on plant phenolic compounds have been found to have faster adhesion rate, affordable cost, excellent availability, and good structural diversity [45,46]. The use of the UV-assisted polymerization of CA also enhances this coating technique. It has thus been demonstrated that UV irradiation can control the polymerization of plant-derived phenolics via induction of reactive oxygen species singlet oxygen (O_2_), superoxide radicals (O_2_^−^) or highly reactive hydroxyl radicals (OH) in the presence of traces of O_2_. Furthermore, the method is simple enough to be used on a larger scale, and the materials used are widely available and simple to set-up.

### 3.2. Surface Topology Analysis

**Figure 2:**
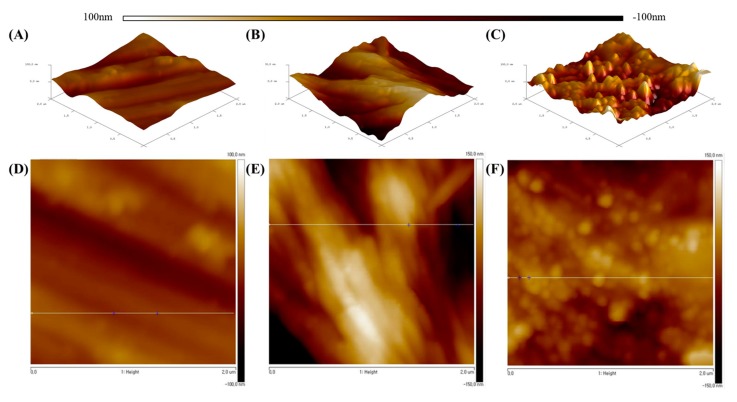
3D and 2D surface AFM images (2 µm × 2 µm) and lateral profiles of each surface: (**A**,**D**) Etched Ti, (**B**,**E**) Ti–PCA, and (**C**,**F**) Ti–PCA–Ag.

We can further determine whether the coating was successfully applied to the sample surface by comparing the roughness of the samples′ surfaces through AFM imaging (2D and 3D). AFM imaging can also indicate the relative surface height of the samples. Examining the 2D and 3D and FESEM images in Figure 2A,D, it can be seen that the surface treated by etching has a thin hatch shape running through a single direction. In Figure 2B,E, the 2D and 3D images show that the coating of PCA on the surface shown an increased surface roughness and a shape resembling the edge of a leaf. As shown in Figure 2C,F, the 2D and 3D AFM images confirm that metallic silver particles are present and had adhered well due to the ridge-like shape formed of the surface of the Ti substrate. The summary of the surface roughness for each sample (etched Ti, Ti–PCA, and Ti–PCA–Ag) are tabulated in Table 1. Showing that the roughness value for each substrate differ with Ti–PCA having the highest roughness value followed by Ti–PCA–Ag. These results further corroborate the initial findings that the coating process was successfully applied by comparing the surface topology difference of each Ti samples.

### 3.3. Surface Chemical Analysis

**Figure 3:**
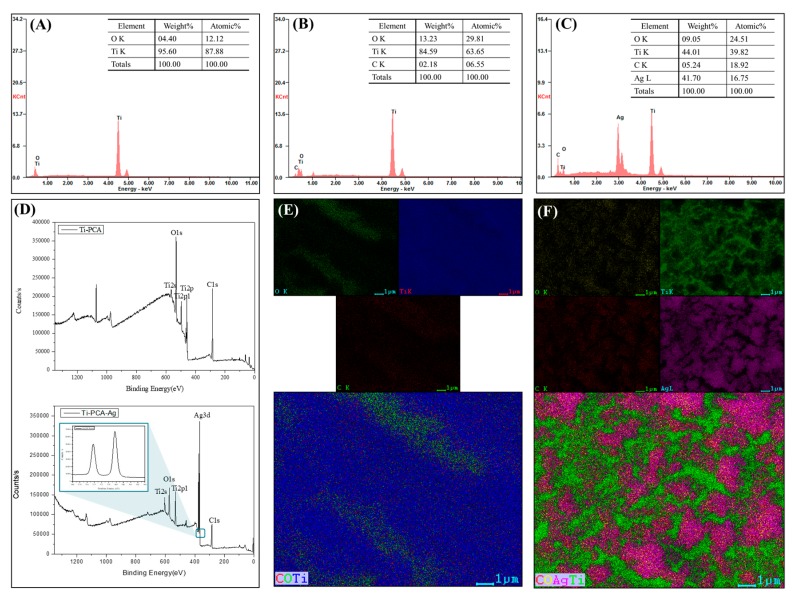
Energy dispersive X-ray spectroscopy (EDS) spectra showing the main components present on the surface of (**A**) Etched Ti, (**B**) Ti–PCA, and (**C**) Ti–PCA–Ag. (**D**) XPS spectra of the Ti–CA (**upper**) and Ti–PCA–Ag (**below**) (inset: XPS spectra Ag3d scan). Mapping of the elemental species on the surface of (**E**) Ti–PCA and (**F**) Ti–PCA–Ag.

EDS, elemental mapping, and XPS were performed in order to verify the FESEM results and to confirm the surface deposition of the PCA and metallic silver on the Ti substrate. As shown in Figure 3A–C, the surface chemical composition changed as the carbon content increased (6.55% and 18%) and oxygen content increased (29.81% and 25.51%) with the Ti–PCA and Ti–PCA–Ag samples, respectively (Figure 3B,C). In contrast to Figure 2A, the chemically etched titanium sample (etched Ti) comprises only of Ti and O elements. The elemental mapping, as seen in Figure 3E,F, verifies the distribution of these elements in the given samples. Ag element is also uniformly distributed on the surface of the samples, as evidenced by the purple color distribution. The XPS spectra are shown in Figure 3D verifying the presence of the metallic silver with a tall Ag3d peak in the survey scan. In addition, the 6 eV peak difference verifies that the Ag element is in a particle zero valence state. As shown in the spectrum, two bands at ca. 368 and 374 eV were observed, which were ascribed to the Ag3d_5/2_ and Ag3d_3/2_ binding energies, respectively.

### 3.4. Physical Surface Characteristic (Wettability)

**Figure 4:**
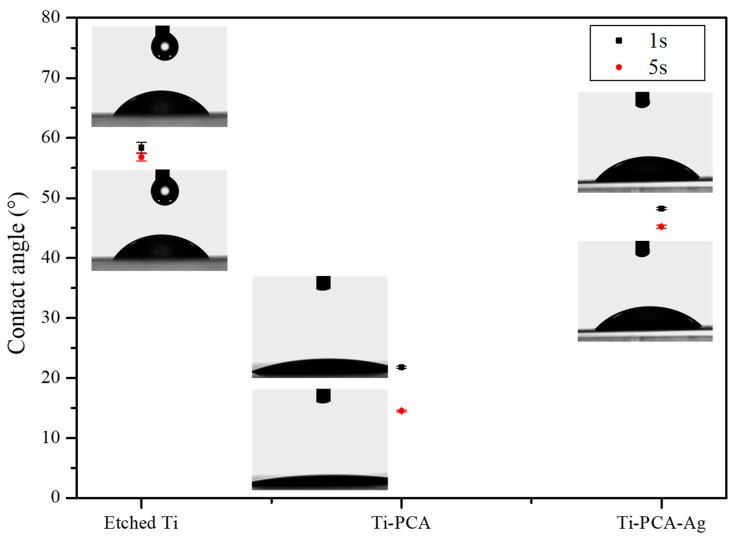
Contact angle values of the Etched Ti (control), Ti–PCA, and Ti–PCA–Ag measured in 1 and 5 s (*n* = 3).

In order to determine the hydrophilic-phobicity of the substrates, contact angle measurements were executed. In principle, droplets with a contact angle of more than 90° are classified as hydrophobic, and those with a contact angle below 90 °C are classified as hydrophilic. A water contact angle with a value close to 0° is superhydrophilic and that with a value above 150° is termed superhydrophobic. Among the many surface modifications of Ti implants, some focused on the increasing surface hydrophilicity because it can influence the bonding strength, the number of proteins bound to a surface, the interactions of the cell with the surface of the implant, the rate of osseointegration, and bacterial adhesion with subsequent biofilm formation [47]. When examining Figure 4, the contact angles of DW after one second were 58.37 ± 0.9°, 21.8 ± 0.2°, 48.2 ± 0.2°, and after 5 s the values are reduced to 56.77 ± 0.61°, 14.5 ± 0.14°, 45.2 ± 0.3° for etched Ti, Ti–PCA, Ti–PCA–Ag, respectively. Ti–PCA turns out to be the most hydrophilic amongst the groups due to the presence of the residual OH^-^ groups on its surface. Ti–PCA–Ag on the other had less OH^−^ groups on its surface due to the reduction of Ag ions to Ag particles and this exhausted some of the PCA coatings, thus making it less hydrophilic than Ti–PCA.

### 3.5. Bacteria Inhibition Test

**Figure 5:**
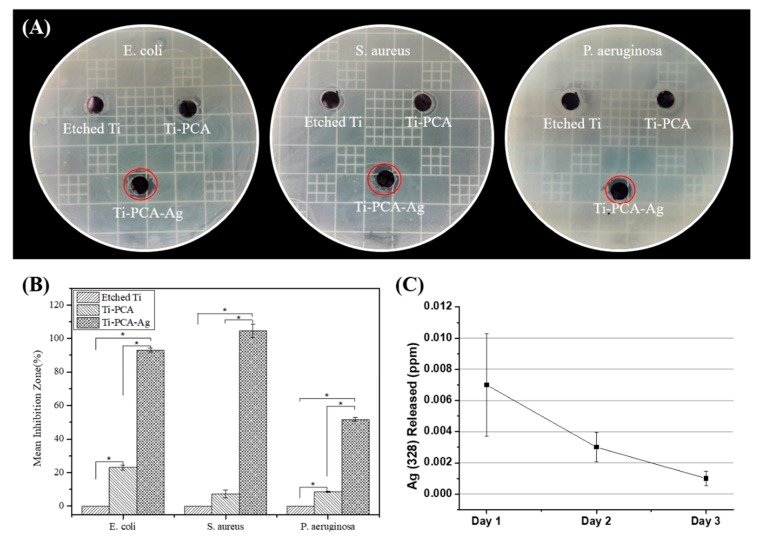
Representative images of antibacterial activities using (**A**) *E. coli* (**left**), *S. aureus (***middle**), and *P. aeruginosa* (**right**) after 24 h after adhered specimens. (**B**) Graph of inhibition zone measured by Image J software (*n* = 3 and * indicates statistical significance (*p* < 0.05) measured by one way ANOVA Tukey test). (**C**) Ag 328 elution from the Ti–PCA–Ag detected via inductively coupled plasma atomic emission spectroscopy (ICP-OES) (*n* = 3).

**Figure 6:**
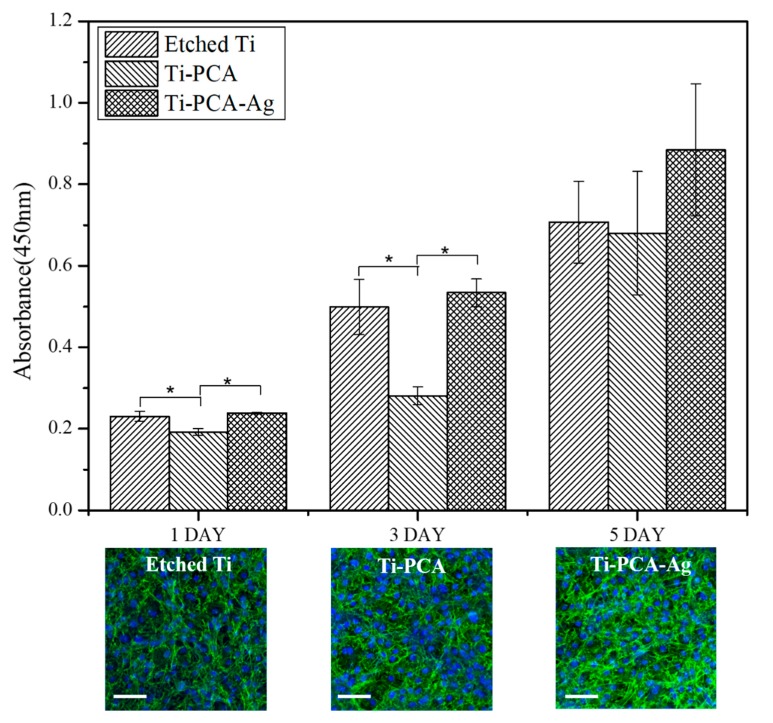
In vitro biocompatibility test using CCK-8 assay of MC-3T3 osteoblast cells. The data are reported as the mean ± standard deviation (*n* = 3 and * indicates statistical significance (*p* < 0.05) measured by one way ANOVA Tukey test) (inset: confocal images of MCT-3T3 cells after five days of incubation) (scale bar = 100 µm).

Until now, increasing the antibacterial properties of Ti are being explored considering that it is one of the most common materials for biomedical implants. In this study, by using three types of bacteria *(E. coli*, *S. aureus*, and *P. aeruginosa*), we demonstrated the antibacterial effect of the coated samples. The result of the test demonstrated that the Ti–PCA–Ag sample has sufficient anti-bacterial efficacy. Especially, among the three strains, *S. aureus* showed the greatest effect due to the Ag particle′s ability to disrupt the cell wall synthesis of gram-positive bacteria. Moreover, it was found that even with only PCA coating, a weak antibacterial effect can be obtained, as reported with other contemporary works, polyphenols can also disrupt bacterial membrane and can lead to increased permeability and susceptibility to damage [41,47]. Moreover, in the case of Ti–PCA–Ag, the effect of silver ions released from metallic Ag particles destroys the biofilm formed by the microorganisms. In addition, the interaction between silver and the protein in the bacteria is considered to be an important mechanism for the antibacterial properties of metallic silver particles. The results in Figure 5A,B shows that the antibacterial effect for all three strains of bacteria is more pronounced on samples containing metallic silver on its surface. Thus, we can attribute the main antibacterial property of coated Ti substrate from the metallic silver and not the PCA coating. The main purpose of the PCA coating is to reduce and immobilize, in-situ the present Ag ions to form metallic silver particles. The mechanism behind the reducing properties of PCA is due to the affinity of metallic ions to the quinone and semiquinone functional moieties of PCA [33]. The ions can agglomerate and form particles on top of the PCA coating creating an enhanced antibacterial surface compared to just using PCA alone. We expect the main antibacterial mechanism of Ti–PCA–Ag is due to the elution of Ag ions from the coating, and this was observed via ICP-OES (Figure 6C). Ag 328 was eluted at the highest concentration during the first day of submersion in distilled water at 0.007 ± 0.0033 ppm. Additionally, as time goes by, the concentration of the Ag 328 in the media has reduced to 0.003 ± 0.0009 ppm (day 2) and 0.0001 ± 0.00004 ppm (day 3). This means that the Ag ions are mostly released after three days and could, therefore, disrupt initial bacterial colonization on the implant substrate.

### 3.6. Biocompatibility on MC-3T3 Pre-Osteoblast Cells

To determine the biocompatibility of the specimens, in vitro cytocompatibility tests were conducted using MC-3T3 pre-osteoblast cell line and the amount of actively metabolic cells were measured using CCK-8 assay (Figure 6). Initially, from the statistical analysis of the results for each specimen, we obtained lesser osteocyte activity for the Ti–PCA sample as a whole for days 1 and 3 compared to Etched Ti and the Ti–PCA–Ag samples. This could be due to the pro-oxidant effect of PCA towards the MC-3T3 cells. However, an upward trend was observed and cell proliferation was increased from days 1, 3, and 5. Moreover, on the fifth day it has almost the same absorbance value as that of the other groups, showing that adhesion of PCA has a positive influence on the cell and cannot be seen as cytotoxic. This is further corroborated by the confocal imaging of MC-3T3 cells after five days of incubation. Cells have adhered and exhibited robust filamentous growth on each of the samples. Ti–PCA–Ag samples also had an upward trend on all time points and comparable to the values of etched Ti. Combining this result with the antibacterial inhibition test, we, therefore, believe that the Ti–PCA–Ag sample is not only suitable for use as an implant material but is also effective in preventing infection and other diseases in peri-implant applications that are still considered to be problematic to date. Furthermore, we anticipate that it will be possible to improve the quality of implants by applying Ti–PCA–Ag modifications to a variety of metallic osteo prosthesis used in situations requiring antimicrobial action.

## 4. Conclusions

In summary, we successfully coated Ti substrates with PCA and metallic silver using a facile UV light assisted method for implant applications. We confirm that the coating process was successful with SEM and AFM analysis. At the same time, we verified the deposition of PCA and metallic silver by confirming the composition of the elements through XPS, EDS, and mapping methods and the physical property (hydrophilicity) of the samples were verified using water contact angle measurements. In addition, Ti–PCA–Ag exhibited antibacterial and biocompatibility properties thus we believe that this method can be used in modifying titanium for osteoprosthesis and other biomedical applications.

## Figures and Tables

**Table 1:** Atomic force microscopy (AFM) analysis survey of the surface roughness of different modified titanium samples.

Samples	Rms (nm)	Ra (nm)	Rmax (nm)
Etched Ti	2.469 ± 1.2	3.280 ± 1.1	10.49
Ti–PCA	25.3845 ± 2.7	39.6855 ± 2.8	163.3
Ti–PCA–Ag	8.6545 ± 0.9	18.2215 ± 1.7	56.67
